# Combination treatment with radiotherapy and a novel oxidative phosphorylation inhibitor overcomes PD-1 resistance and enhances antitumor immunity

**DOI:** 10.1136/jitc-2019-000289

**Published:** 2020-06-24

**Authors:** Dawei Chen, Hampartsoum B Barsoumian, Grant Fischer, Liangpeng Yang, Vivek Verma, Ahmed I Younes, Yun Hu, Fatemeh Masropour, Katherine Klein, Christopher Vellano, Joseph Marszalek, Michael Davies, Maria Angelica Cortez, James Welsh

**Affiliations:** 1 Department of Radiation Oncology, Shandong Cancer Hospital and Institute, Shandong First Medical University and Shandong Academy of Medical Sciences, Jinan, China; 2 Radiation Oncology, University of Texas MD Anderson Cancer Center, Houston, Texas, USA; 3 Department of Cancer Biology, The University of Texas MD Anderson Cancer Center, Houston, United States; 4 Department of Melanoma Medical Oncology, The University of Texas MD Anderson Cancer Center, Houston, United States; 5 Department of Translational Molecular Pathology, The University of Texas MD Anderson Cancer Center, Houston, United States; 6 Department of Radiation oncology, Allegheny General Hospital, Pittsburgh, United States; 7 Translational Research to Advance Therapeutics and Innovation in Oncology Platform, The University of Texas MD Anderson Cancer Center, Houston, United States; 8 Therapeutics Discovery Division, The University of Texas MD Anderson Cancer Center, Houston, United States; 9 Institute for Applied Cancer Science, The University of Texas MD Anderson Cancer Center, Houston, TX, United States; 10 Department of Systems Biology, The University of Texas MD Anderson Cancer Center, Houston, Houston, TX, United States

**Keywords:** radiotherapy, immunology, tumor

## Abstract

**Background:**

Despite outstanding responses to anti-PD-1 agents in a subset of non-small cell lung cancer (NSCLC) patients, approximately 80% of patients fail to have prolonged favorable response. Recent studies show that tumor cell oxidative metabolism is a barrier to PD-1 immunotherapy and radiotherapy could overcome PD-1 resistance, so it is urgent to determine if combination treatment with radiotherapy and a novel oxidative phosphorylation (OXPHOS) inhibitor (IACS-010759) is an effective strategy against PD-1 resistance in NSCLC.

**Methods:**

The antitumor effect of this combinational treatment was evaluated in vitro and in vivo. For in vivo experiments, we treated 129Sv/Ev mice with anti-PD1-sensitive and anti-PD1-resistant 344SQ NSCLC adenocarcinoma xenografts with oral IACS-010759 combined with radiotherapy (XRT). In vitro experiments included PCR, seahorse bioenergetic profiling, flow cytometry phenotyping, and clonogenic survival assay.

**Results:**

In the current study, we found that our PD-1-resistant model utilized OXPHOS to a significantly greater extent than the PD-1-sensitive model and XRT increased OXPHOS in vitro and in vivo. Thus, we explored the effect of the novel OXPHOS inhibitor IACS-010759 on PD-1-resistant NSCLC in an effort to overcome XRT-induced immunosuppression and maximize response to PD-1. Additionally, combined XRT and IACS-010759 promoted antitumor effects in the PD-1-resistant model, but not in the sensitive model. After elucidation of the most optimal dose/fractionation scheme of XRT with IACS-010759, the combinatorial therapy with this regimen did not increase the abscopal antitumor effect, although IACS-010549 did not decrease CD45+, CD4+, and CD8+ immune cells. Finally, triple therapy with IACS-010759, XRT, and anti-PD-1 promoted abscopal responses and prolonged survival time.

**Conclusion:**

OXPHOS inhibition as part of a combinatorial regimen with XRT is a promising strategy to address PD-1-resistant NSCLC, and this combination is being tested clinically.

## Introduction

It is well known that cancer cells have upregulated glycolysis compared with non-cancer cells, which may lead to the downregulation of oxidative phosphorylation (OXPHOS). However, recent studies indicate that mitochondrial OXPHOS is not impaired, but also more activated.^1^ Others have demonstrated that mitochondrial OXPHOS could be a target for cancer treatment and enhancement of immunotherapy outcomes, which currently remain low in metastatic non-small cell lung cancer (NSCLC).^2 3^ Therefore, it is valuable to explore if inhibition of mitochondrial OXPHOS could overcome PD-1 resistance in metastatic NSCLC.

Recently, it has been reported that mitochondrial inhibition via IACS-010759 overcame MAPK inhibitor-resistance in melanoma,^4^ and metformin plus tyrosine kinase inhibitors (TKIs) significantly increased treatment efficacy compared with TKIs alone in epidermal growth factor receptor-mutated NSCLC,^5^ indicating that combining metabolic therapy with biologic targeted therapy is an effective way to control cancer growth. IACS-010759 and metformin inhibit complex I of the electron transport chain. Thus, these findings may challenge the Warburg effect, which is the traditional viewpoint that tumor cells preferentially engage in glycolysis for ATP production, as opposed to normal tissues, which primarily use^6–9^ OXPHOS. Recent studies in several cancers (pancreatic adenocarcinoma, melanoma, and leukemias) have also challenged the Warburg effect by reporting that mitochondrial metabolism was not impaired but actually increased in tumor cells. These findings provide a rationale to use OXPHOS inhibitors to target certain tumors relying on OXPHOS to meet their bioenergetic needs.

OXPHOS is impacted by external influences such as radiotherapy. Following radiotherapy (XRT), OXPHOS is upregulated and assists the remaining viable cancer cells with metabolic demands.^7 8^ In this manner, XRT can have deleterious effects on the tumor microenvironment, although it carries the unique advantage of overcoming PD-1 resistance by releasing tumor-associated antigens, activating type 1 interferon (IFN) signaling, and inducing antitumor immunity.^9^

IACS-010759 is a novel and specific inhibitor for mitochondrial complex 1 (thus inhibiting OXPHOS), and it showed promising treatment efficacy in translational models of hematologic tumors, SWI/WNF-mutated lung cancer, and metastatic melanoma.^10–12^ These results led to the construction of phase 1 clinical trials in leukemia (NCT02882321) and metastatic solid tumors (NCT03291938), which are ongoing.

In the current study, our goals are to assess differential metabolic parameters in PD-1-sensitive and PD-1-resistant NSCLC models, evaluate the combined effect of XRT and IACS-010759 in both models, explore the effect of combination therapy on abscopal responses, and provide mechanistic insight into these observations.

## Methods

### Cell lines and irradiation

The PD-1-sensitive 344-SQ murine NSCLC adenocarcinoma cell line was a gift from Dr Jonathan M. Kurie at MD Anderson. The cells were cultured in RPMI-1640 medium with 10% fetal bovine serum and 1% antibiotics at 37°C in a humidified 5% CO_2_ incubator. The PD-1-resistant 344-SQ cell line^9^ was developed from parental 344-SQ PD-1-sensitive cell lines treated with anti-PD-1 in 129Sv/Ev mice as per previous studies.^13 14^ Both of these cell lines were verified by DDC Medical by short-tandem-repeat DNA fingerprinting. Cells were irradiated at room temperature with a Mark I ^137^Cs irradiator at a dose rate of 3 Gy/min.

### PCR array analysis of genes involved in mitochondrial OXPHOS

Total RNA was isolated from PD-1-sensitive and PD-1-resistant cells with the RNeasy Mini Kit (Qiagen, Cat# 74106). RNA from tumors was extracted after being frozen at −80°C and homogenized in Trizol (Invitrogen, Cat# 10296028). RNA quality control was performed with Nanodrop 2000. Next, mRNA was reverse-transcribed by the iScript Reverse Transcription Supermix for reverse transcription quantitative PCR (Bio-Rad, Cat#1708841) and loaded into a customized 384-well PCR plate (Bio-rad, Cat No.10034696) with a panel of mitochondrial OXPHOS-related genes. Then, multiple quantitative PCR assays were done with a CFX96 Touch Real-Time PCR Detection System with SYBR Green (Life Technologies).

### Seahorse bioenergetic profiling

A Seahorse XFe96 Bioanalyzer (Agilent) was used according to the manufacturer’s instructions. Briefly, cells were plated in a 96-well Seahorse XF Cell Culture Microplate at a density of 25,000/well in 100 µL of RPMI-1640 medium with 10% fetal bovine serum and 1% antibiotics and incubated for 12 hours at 37°C under 5% CO_2_. For the Mito Stress Test, media was then removed and replaced with minimal, unbuffered Dulbecco modified Eagle medium supplemented with 5 mM glucose, 1 mM pyruvate, and 2 mM glutamine for a 1 hour CO_2_-free incubation at 37°C. Basal oxygen consumption rate (OCR) was recorded prior to treatment with 1.5 µM oligomycin, 0.5 µM FCCP, and 0.5 µM rotenone/antimycin A for three cycles of 2 min mixes, 2 min wait times, and 3 min measure times. For the Glycolysis Stress Test, media was removed and replaced with minimal, unbuffered Dulbecco modified Eagle medium supplemented with 1 mM glutamine for a 1 hour CO_2_-free incubation at 37°C. Basal extracellular acidification rate (ECAR) was recorded prior to treatment with 10 mM glucose, 1.5 µM oligomycin, and 50 mM 2-deoxyglucose for three cycles of 2 min mixes, 2 min wait times, and 3 min measure times. All these experiments were normalized to cell number in case of potential confounding impact of cell death arising from radiation.

### Clonogenic survival assay

Cells were seeded into six-well plates with different densities (300 cells/well for 0 Gy, 500 cells/well for 2 Gy, 1500 cells/well for 4 Gy, and 3000 cells/well for 6 Gy) and preadministered with IACS-010759 in prespecified concentrations for 48 hours. Irradiation was performed 4 hours later. Cells were then incubated and monitored for 8 days at 37°C in a humidified chamber containing 5% CO_2_.

### In vivo experiments

The details and protocol to establish the murine tumor model followed our previous studies.^8 13^ Briefly, eight to 10-week-old female 129Sv/Ev mice were used in the current study. For the PD-1-sensitive one-tumor model, tumors were established with subcutaneous injections of 0.5×1 0^6^ 344 SQ PD-1-sensitive cell line; for the PD-1-resistant one-tumor model, tumors were established with 1×1 0^5^ 344 SQ PD-1 resistant cell line. For the PD-1 resistant two-tumor model, the primary (irradiated) tumor was established with 1×1 0^5^ 344 SQ PD-1 resistant cells and 0.2×10^5^ for abscopal (non-irradiated) tumor 3 days later. IACS-010759 was orally administered at 7.5 mg/kg every day until the endpoint; α-PD-1 monoclonal antibody (Bio X-cell; Cat No. BE0273) was given intraperitoneally at 200 µg on days 5, 9, 13, and 17; radiation was administered to the primary tumors on days 7, 8, and 9. All animal work was conducted in accordance with Institutional Animal Care and Use Committee (policies at MD Anderson Cancer Center).

### Flow cytometry phenotyping

Tumor-infiltrating lymphocyte isolation was performed as previously described.^9^ Cells were blocked with antimouse CD16/32 for 30 min at 4°C and then stained with CD45 APC (Cat #187357), CD4 APC-Fire 750 (Cat #244105), CD8 PercpCy5.5 (Cat #277115), Foxp3 Alexa488 (Cat #227489), Granzyme B Pacific-blue (Cat #267707), Gr1 BV510 (Cat #238839), CD11b Alexa700 (Cat #259438), CD38 PE-Cy7 (Cat #216741), and CD206 PE (Cat #225355) from BioLegend. Samples were run on a Gallios (BD Biosciences) flow cytometer and analyzed with Kaluza Analysis Software.

### Statistical analysis

Statistical analyzes were conducted using GraphPad Prism software (V.8.0). Tumor growth curves were compared using two-way analysis of variance. Survival was analyzed using the Kaplan-Meier method and compared using log-rank tests. Student t tests were used to compare bar charts of various treatment conditions. Statistical significance was defined as p<0.05.

## Results

### The PD-1-resistant model has a more aerobic phenotype than the PD-1-sensitive model

**Figure 1:**
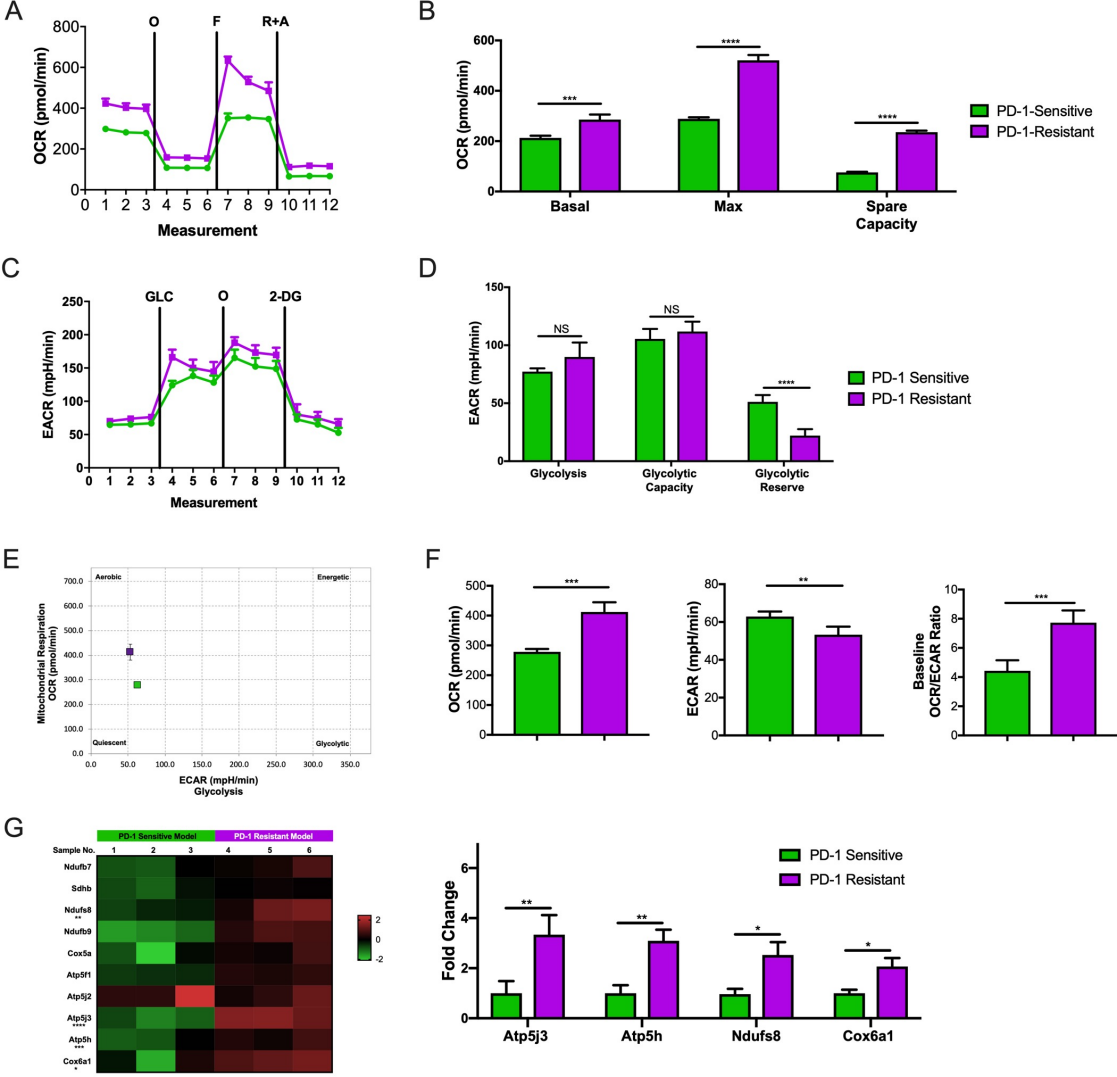
PD-1-resistant model was characterized by a more aerobic phenotype than the PD-1-sensitive model. (A) Seahorse Mito Stress Test analysis showing the baseline, oligomycin-inhibited (O), FCCP-activated (F), and rotenone/antimycin-inhibited (R+A) oxygen consumption rate (OCR) in PD-1-sensitive and PD-1-resistant 344-SQ NSCLC cells. Data are an average of quadruplicates. (B) Basal respiration (t-test, p=0.0004), maximal respiration (t-test, p<0.0001), and spare respiratory capacity (t-test, p<0.0001) were calculated from the Mito Stress Test data in (A). All three variables were significantly increased in the resistant cells compared with the sensitive cells. Data are presented as mean with SD (n=4 wells/group). (C) Seahorse Glycolysis Stress Test analysis showing the baseline, glucose-stimulated (GLC), oligomycin-activated (O), and 2-deoxyglucose-inhibited (2-DG) extracellular acidification rate (ECAR) in PD-1-sensitive and PD-1-resistant 344-SQ NSCLC cells. Data are an average of quadruplicates. (D) Glycolysis, glycolytic capacity, and glycolytic reserve capacity were calculated from the Glycolysis Stress Test data in (E). Glycolysis and glycolytic capacity did not differ between PD-1-resistant and PD-1-sensitive cells, but resistant cells were characterized by a significantly lower glycolytic reserve (t-test, p=0.0004) capacity than sensitive cells. Data are presented as mean with SD (n=4 wells/group). (E) Baseline OCR and baseline ECAR from the Mito Stress Test data from (A) were plotted against each other to yield a cell energy phenotype diagram for the sensitive and resistant cells. The diagram labels cells as aerobic, quiescent, glycolytic, or energetic on the basis of their OCR/ECAR ratios. The resistant cells were characterized by a more aerobic phenotype than sensitive cells. (F) Baseline OCR, baseline ECAR, and baseline OCR/baseline ECAR ratio were calculated for the resistant and sensitive cell lines on the basis of the Mito Stress Test data from (A). Resistant cells were characterized by significantly higher baseline OCR (t-test, p=0.0002) and significantly lower baseline ECAR (t-test, p=0.0084) than sensitive cells. The OCR/ECAR ratio (t-test, p=0.001) was nearly twice as large in resistant cells as in sensitive cells. Data are presented as mean with SD. (G) PCR assay heat map analysis showing that the PD-1-resistant model has more OXPHOS-related gene expression; expression of Atp5j3 (t-test, p=0.006), Atp5h (t-test, p=0.008), Ndufs8 (t-test, p=0.022), and Cox6a1 (t-test, p=0.039) were statistically higher in the resistant model than in the sensitive model (n=3 wells/group; data presented as mean with SD). Data in B, D, F, and G were analyzed by t test (NS, not significant, *p<0.05, **p<0.01, ***p<0.001, ****p<0.0001). All this data were performed twice under the same schedule to confirm the results, and “N” means different wells in each group from one representative experiment. NSCLC, non-small cell lung cancer; OXPHOS, oxidative phosphorylation.

We performed the Seahorse Mito Stress Test to acquire baseline, oligomycin-inhibited, FCCP-activated, and rotenone/antimycin-inhibited OCR values in the PD-1-sensitive and PD-1-resistant 344-SQ NSCLC cell lines growing in vitro (figure 1A). Basal respiration (p=0.0006), maximal respiration (p<0.0001), and spare respiratory capacity (p<0.0001) were significantly higher in the PD-1-resistant model than in the PD-1-sensitive model (figure 1B). The Seahorse Glycolysis Stress Test captured baseline, glucose-stimulated, oligomycin-activated, and 2-deoxyglucose-inhibited ECAR values in these two models (figure 1C). Glycolysis and glycolytic capacity did not differ between PD-1-resistant and PD-1-sensitive cells, but resistant cells were characterized by a significantly lower glycolytic reserve capacity than sensitive cells (p<0.0001; figure 1D). We then plotted the baseline OCR and baseline ECAR values from the Mito Stress Test data against each other to define the metabolic phenotype of the PD-1-resistant and PD-1-sensitive models. As shown in figure 1E, the PD-1-resistant model was characterized by a more aerobic phenotype than the PD-1-sensitive model. PD-1-resistant cells were also characterized by significantly higher baseline OCR values (p=0.002) and baseline OCR/baseline ECAR ratio (p=0.001) but significantly lower baseline ECAR values (p=0.008) than PD-1-sensitive cells (figure 1F). Confirmatory PCR assay analysis showed that OXPHOS-related genes were upregulated in PD-1-resistant model comparing to the PD-1-sensitive model (figure 1G).

To better present and validate our findings with the 344SQ PD1-sensitive and PD1-resistant models, we picked two more cell lines with different sensitivities to anti-PD1. The first-cell line was Lewis Lung Carcinoma (LLC) which is immunologically cold and has low sensitivity to PD-1 antibody. The second was PANC-2 cell line, which is sensitive to PD-1 antibody, and can be regarded as PD-1-sensitive model. We then performed the Seahorse Mito Stress Test to acquire baseline, oligomycin-inhibited, FCCP-activated, and rotenone/antimycin-inhibited OCR values (online supplementary figure 1A). Basal respiration (p=0.02), maximal respiration (p=0.0004), and spare respiratory capacity (p=0.002) were significantly higher in the LLC cell line (PD-1-resistant) than in the PANC-2 cell line (PD-1-sensitive) (online supplementary figure 1B). The Seahorse Glycolysis Stress Test captured baseline, glucose-stimulated, oligomycin-activated, and 2-deoxyglucose-inhibited ECAR values in these two models (online supplementary figure 1C). Glycolysis and glycolytic capacity did not differ between LLC and PANC-2 cell lines in glycolysis, glycolytic capacity, and glycolytic reserve (p>0.05, respectively; online supplementary figure 1D).

10.1136/jitc-2019-000289.supp1 Supplementary data

Together, these profiling results indicate that the PD-1-resistant cells utilized OXPHOS significantly more than PD-1-sensitive cells did. Moreover, the PD-1-resistant cells increased their utilization of OXPHOS, but not glycolysis.

### XRT increases OXPHOS in the PD-1-resistant 344-SQ model in vitro and in vivo

**Figure 2:**
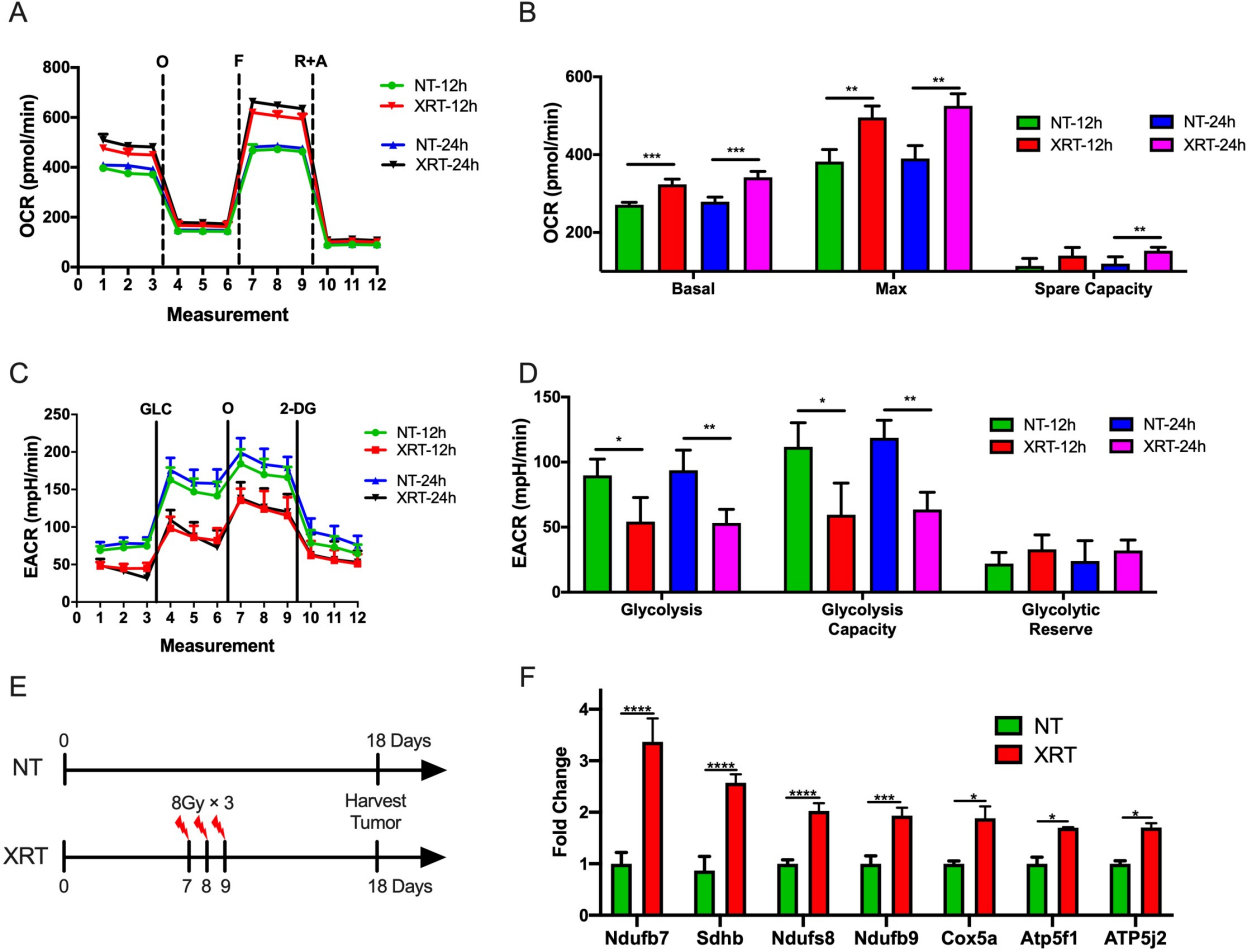
Radiotherapy (XRT) increases oxidative phosphorylation (OXPHOS) and decreases glycolysis in the PD-1-resistant model. (A) Seahorse Mito Stress Test analysis showing oligomycin-inhibited (O), FCCP-activated (F), and rotenone/antimycin-inhibited (R+A) oxygen consumption rate (OCR) in the PD-1-resistant cell line 12 and 24 hours after XRT in 8 Gy. (B) XRT significantly increased basal respiration (t-test, p=0.0004, 12 hours after XRT; p=0.0006, 24 hours after XRT), maximal respiration (t-test, p=0.002, 12 hours after XRT; p=0.001, 24 hours after XRT), and spare respiratory capacity (t-test, p=0.02, 24 hours after XRT) compared with control (n=4 wells/group). (C) Seahorse Glycolysis Stress Test analysis showing glucose-stimulated (GLC), oligomycin-activated (O), and 2-deoxyglucose-inhibited (2-DG) extracellular acidification rate (ECAR) in the PD-1-resistant cell line 12 hours after XRT. (D) XRT decreased glycolysis (t-test, p=0.02, 12 hours after XRT; p=0.005, 24 hours after XRT) and glycolytic capacity (t-test, p=0.014, 12 hours after XRT; p=0.001, 24 hours after XRT) compared with control (n=4 wells/group). (E) Illustration diagram for PCR in vivo. (F) PCR results showed that XRT increased the expression of OXPHOS-related Ndufb7 (t-test, p<0.0001), Sdhb (t-test, p<0.0001), Ndufs8 (t-test, p<0.0001), Ndufb9 (t-test, p=0.0004), Cox5a (t-test, p=0.013), Atp5f1 (t-test, p=0.018), and Atp5j2 (t-test, p=0.032) (n=3 wells/group). Data are presented as mean with SD (NS, not significant, *p<0.05, **p<0.01, ***p<0.001, ****p<0.0001). All these data were done twice under the same schedule to confirm the results, and “N” means different wells in each group from one representative experiment. NT, no treatment.

It has been previously shown that the PD-1-resistant 344-SQ model was more sensitive to radiation than the PD-1-sensitive model.^9^ Therefore, we sought to explore whether radiation was associated with metabolic effects in the two models. First, the Seahorse Mito and Glycolysis Stress Tests were performed after XRT of cells growing in vitro. To imitate the clinical practice in an iRT clinical trial,^15^ we picked XRT in 8 Gy dose with different time points (12 and 24 hours after XRT) and used another two schemes (4 and 6 Gy) to validate this phenotype. Interestingly, XRT (8 Gy) significantly increased basal respiration (p=0.0004, 12 hours after XRT; p=0.0006, 24 hours after XRT), maximal respiration (p=0.002, 12 hours after XRT; p=0.001, 24 hours after XRT), and spare respiratory capacity (p=0.02, 24 hours after XRT) in the PD1-resistant 344-SQ model (figure 2A, B). Additionally, XRT decreased glycolysis (p=0.02, 12 hours after XRT; p=0.005, 24 hours after XRT) and glycolytic capacity (p=0.014, 12 hours after XRT; p=0.001, 24 hours after XRT) compared with control (figure 2C, D). Another two schemes (4 and 6 Gy) also validated this phenotype 12 hours after XRT. XRT significantly increased basal respiration (p=0.0005 in the 6 Gy group), maximal respiration (p=0.011 in the 4 Gy group; p<0.0001 in the 6 Gy group), and spare respiratory capacity (p=0.019 in the 6 Gy group) in the PD1-resistant 344-SQ model (online supplementary figure 2A and B). Additionally, XRT decreased glycolysis (p=0.0003 in the 6 Gy group) and glycolytic capacity (p<0.0001 in the 6 Gy group) compared with control (online supplementary figure 2C and D).

The experiment was also performed in the PD-1-sensitive cells, including the Seahorse Mito Stress Test (online supplementary figure 3A and B) and Glycolysis Stress Test (online supplementary figure 3C and D). In contrast to the PD-1 resistant model, no significant differences in OXPHOS or glycolysis were observed with irradiation. Taken together, in addition to having higher OXPHOS at baseline, the PD1-resistant cells showed a differential induction of OXPHOS following radiation compared with the PD-1-sensitive NSCLC cells in vitro.

To further explore this observation, the effects of XRT on OXPHOS in the PD-1-resistant line in vivo was evaluated (figure 2E). Briefly, 1×10^5^ PD-1 resistant 344SQ cells were injected s.c. in the left leg, and XRT (8 Gy×3 fractions) was administered to the tumors on days 7, 8, and 9. Tumor tissues were harvested on day 18, and RNA was isolated for PCR. This analysis showed that radiation increased the expression of multiple OXPHOS-related genes, including Ndufb7 (p<0.0001), Sdhb (p<0.0001), Ndufs8 (p<0.0001), Ndufb9 (p=0.0004), Cox5a (p=0.013), Atp5f1 (p=0.018), and Atp5j2 (p=0.032) (figure 2F).

### Combining IACS-010759 with XRT promotes antitumor effects in the PD-1-resistant model

**Figure 3:**
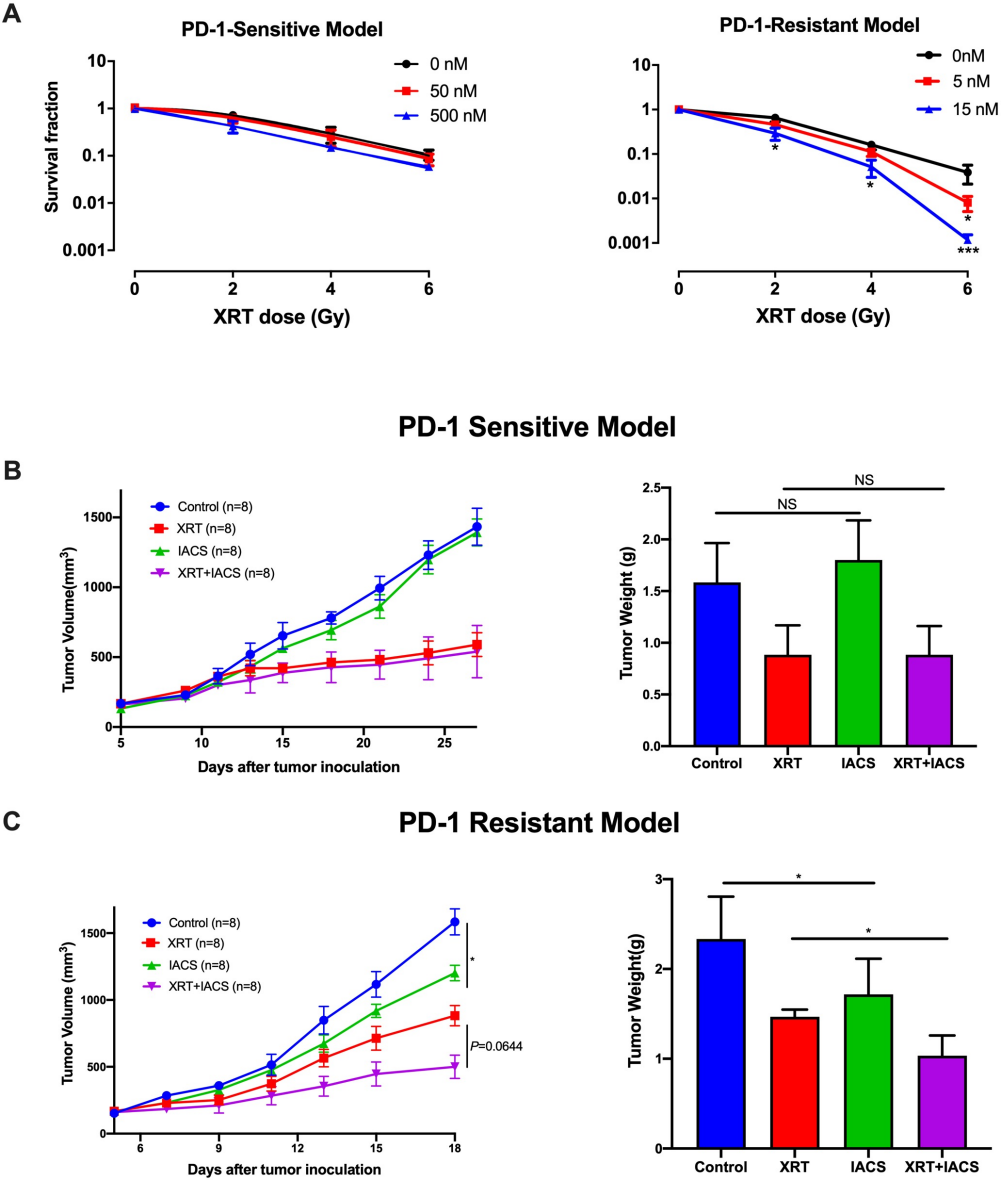
Combining radiotherapy (XRT) with IACS-010759 promotes antitumor effects in the PD-1-resistant model. The clone formation assay was used to test this combination regimen in vitro. (A) In the PD-1-sensitive model, IACS-010759 did not increase local control when added to XRT. In the PD-1-resistant model, IACS-010759 combined with XRT had a synergistic antitumor effect, inhibiting clone formation at doses of 5 nM IACS-010759 with 6 Gy XRT (t-test, p=0.04) and 15 nm IACS-010759 with 2 Gy XRT (t-test, p=0.032), 4 Gy XRT (t-test, p=0.01), and 6 Gy XRT (t-test, p=0.0008) (n=3 wells/group). (B) IACS-010759 did not inhibit tumor growth in vivo and did not increase local control with XRT in the PD-1-sensitive model. NT, no treatment (control) (n=8 mice/group). (C) IACS-010759 significantly inhibited tumor growth in vivo compared with control (two-way ANOVA, p=0.023), and IACS-010759 combined with XRT increased local control compared with XRT alone, although this was not statistically significant (two-way ANOVA, p=0.064). Tumor weight was significantly lower after IACS-010759 alone compared with control (t-test, p=0.031) and XRT alone (t-test, p=0.044). Data are presented as mean with SD (NS, not significant, *p<0.05) (n=8 mice/group). ANOVA, analysis of variance.

Given the findings shown in figures 1 and 2, we next wanted to evaluate whether adding IACS-010759 to XRT could increase the antitumor effects. First, the clonogenic assay was performed to test this combination regimen in vitro (figure 3A) and found enhanced inhibition clonogenic capacity in PD-1-resistant cell line treated with 5 nM IACS-010759 with 6 Gy XRT (p=0.04) and 15 nM IACS-010759 with 2 Gy XRT (p=0.032), 4 Gy XRT (p=0.01), and 6 Gy XRT (p=0.0008), but no significant difference was detected in the PD-1-sensitive model. To test whether IACS-010759 reverts resistant cells to sensitive metabolic profile, Seahorse Mito Stress Test was performed to detect the metabolic effect of IACS-010759 on PD1-sensitive and PD1-resistant cells and indicated that after IACS-010759 treatment (50 nM) 12 hours in vitro, these two cell lines showed similar mitochondrial OXPHOS phenotype in basal respiration (p=0.74), maximal respiration (p=0.62), and spare respiratory capacity (p=0.9) (online supplementary figure 4A and B).

For subsequent in vivo testing, mice were implanted subcutaneously with PD-1-sensitive or PD-1-resistant cells and randomized into one of four groups: control, XRT (12 Gy ×3) alone, IACS-010759 alone, or XRT +IACS-010759. All mice were euthanized and tumors harvested when any tumor reached 1500 mm^3^ in a given group. The trial lasted 28 days in the sensitive model and 18 days in the resistant model. Consistent with the in vitro data, IACS-010759 did not exhibit additional effects on tumor growth compared with XRT alone in the PD-1-sensitive model (figure 3B). However, in the PD-1-resistant model, IACS-010759 significantly inhibited tumor growth compared with control (p=0.023), and combined therapy increased local control compared with XRT alone, although this was not statistically significant (p=0.064; figure 3C). Similar outcomes were observed for tumor weight (p=0.031 and p=0.044, respectively; figure 3C).

In summary, these data illustrate that IACS-010759 significantly inhibited tumor growth and seemed to increase the efficacy of XRT in the PD-1-resistant model.

### 8 Gy×3 might be the optimal XRT dose and fractionation scheme to combine with IACS-010759

**Figure 4:**
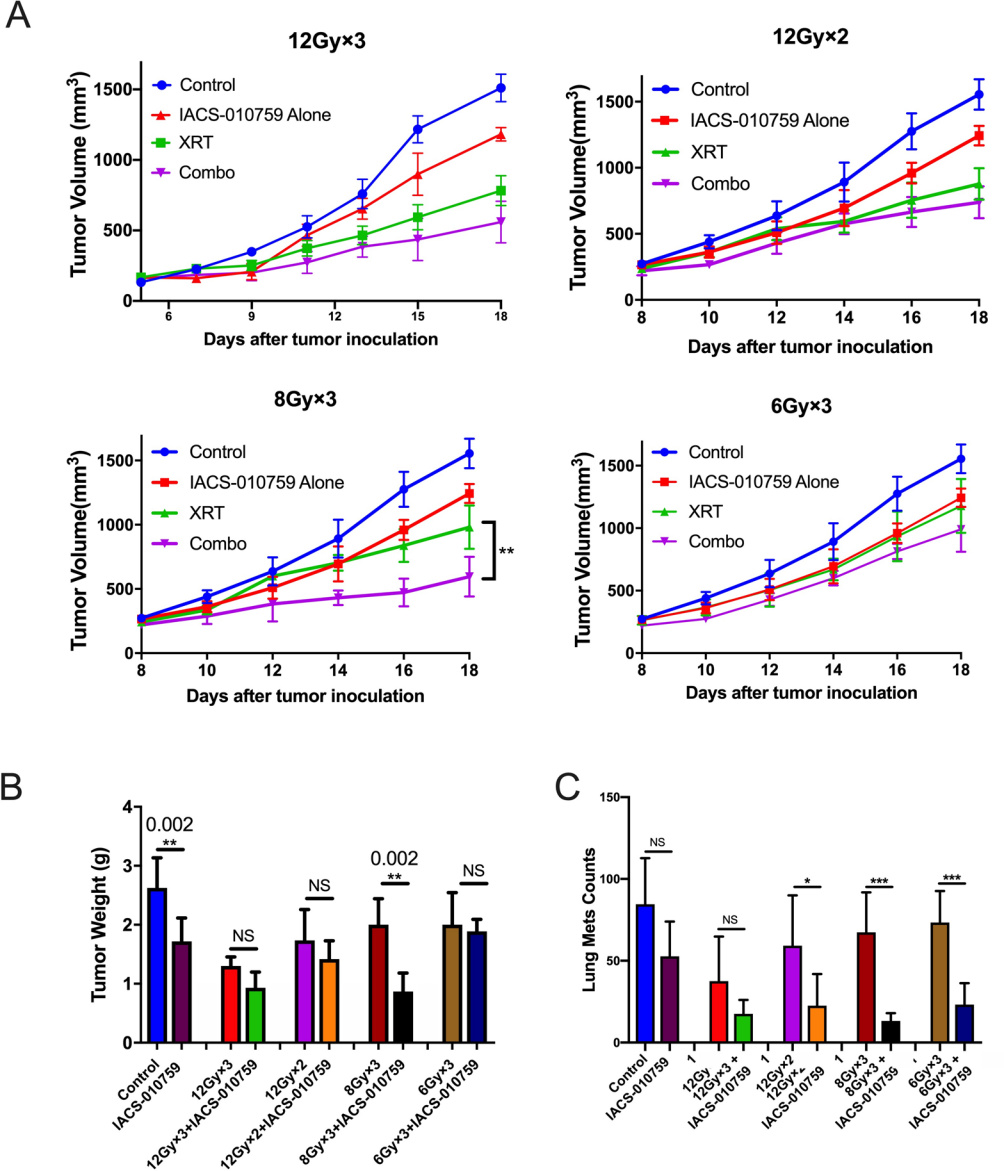
8 Gy×3 might be the most optimal radiotherapy (XRT) dose and fractionation scheme when combined with IACS-010759. Mice (six per group) were injected subcutaneously with 1×10^5^ PD-1-resistant cells into the right leg and tumors were harvested on day 18. Three radiation schemes were chosen for comparison: 12 Gy×2 fractions, 8 Gy×3 fractions, and 6 Gy×3 fractions. IACS-010759 was administrated as per the protocol described in the text. Thus, the experiment had eight groups: control, IACS-010759 alone, 12 Gy×2 XRT, 8 Gy×3 XRT, 6 Gy×3 XRT, 12 Gy×2 XRT combined with IACS-010759, 8 Gy×3 XRT combined with IACS-010759, and 6 Gy×3 XRT combined with IACS-010759. (A) Data are presented according to XRT scheme. Only 8 Gy×3 combined with IACS-010759 showed significantly increased efficacy compared with XRT alone (two-way ANOVA, p=0.003). (B) For tumor weight, 8 Gy×3 combined with IACS-010759 was more optimal than 12 Gy×2 combined with IACS-010759 (t-test, p=0.012) or 6 Gy×3 combined with IACS-010759 (t-test, p<0.0001). (C) Representative images showing tumor burden in each treatment group. (D) Lung metastases on day 18 in each group. (E) Representative images of lung metastases on day 18 in each group. Data are presented as mean with SD (*p<0.05, **p<0.01, **p<0.01, ****p<0.0001). ANOVA, analysis of variance; NS, not significant.

To identify the optimal dose and fractionation scheme of XRT in combination with IACS-010759, we tested three schedules in the one-tumor mouse model (figure 4A): 12 Gy×3 fractions (higher stereotactic dose which previously found to overcome anti-PD1 resistance^9 14^), 12 Gy×2 fractions (higher stereotactic dose), 8 Gy×3 fractions (median stereotactic dose), and 6 Gy×3 fractions (lower stereotactic dose). We found that 8 Gy×3 with IACS-010759 produced the greatest local control (p=0.004), whereas 12 Gy×3, 12 Gy×2, and 6 Gy×3 did not significantly decrease tumor growth compared with XRT alone.

Similar findings were observed for tumor weights harvested on day 18 showed that only 8 Gy×3 with IACS-010759 significantly decreased tumor weight compared with 8 Gy×3 alone (p=0.002, figure 4B). Additionally, XRT combined with IACS-010759 significantly decreased the number of lung metastases compared with XRT alone in 12 Gy×2 group (p=0.03), 8 Gy×3 group (p=0.0001), and 6 Gy×3 group (p=0.0001, figure 4C).

We then explored whether XRT and IACS-010759 could induce abscopal responses in the two-tumor model. We chose two XRT regimens to combine with IACS-010759: 8 Gy×3 (based on the above data) and 12 Gy×3 (previously found to overcome PD-1 resistance and induce an abscopal effect). Neither regimen, when combined with IACS-010759, induced an abscopal response (online supplementary figure 5A). However, combination therapy significantly increased survival compared with IACS-010759 alone (p=0.028 for 8 Gy×3 and p=0.014 for 12 Gy×3; online supplementary figure 5B).

### IACS-010759 decreases radiation-induced regulatory T cells and increases activated CD8+ T cells

**Figure 5:**
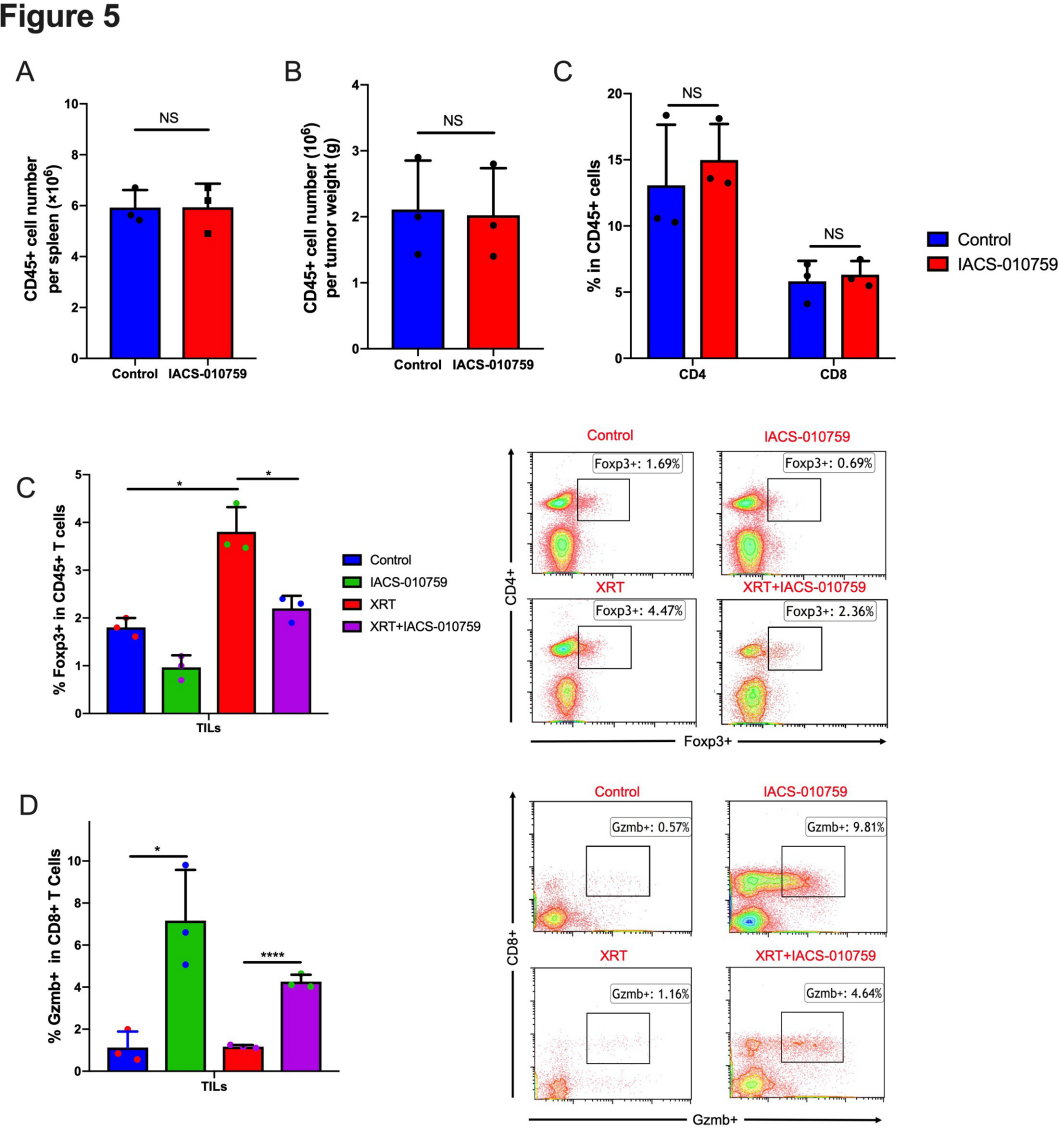
IACS-010759 decreases radiation-induced regulatory T cells and increases activated CD8+ T cells. We observed no obvious difference in the total number of (A) CD45+ cells in spleen (t-test, p=0.68) and (B) tumor (t-test, p=0.652) or (C) CD4+ (t-test, p=0.41) and CD8+ (t-test, p=0.892) cells (tumor-infiltrating lymphocytes; TILs) isolated from tumors after IACS-010759 administration. (E) IACS-010759 can decrease radiation-induced T_regs_ (t-test, p=0.0076, radiotherapy (XRT) alone compared with XRT combined with IACS-010759). (E) IACS-010759 increased Gzmb+ T cells compared with control (t-test, p=0.015), and the combination of XRT with IACS-010759 significantly increased Gzmb+ T cells compared with XRT alone (t-test, p<0.0001). Data are presented as mean with SD (*p<0.05, **p<0.01, ****p<0.0001). Data in this figure were performed twice under the same schedule to confirm the results. Data are presented as mean with SD (*p<0.05, **p<0.01, **p<0.01, ****p<0.0001). NS, not significant.

To assess the status of immune cells when IACS-010759 was administered, we isolated splenocytes and tumor-associated immune cells for flow cytometry analysis on day 21 after tumor establishment. IACS-010759 did not decrease CD45+ cells in spleen (figure 5A) and tumor (figure 5B) and total CD4+ and CD8+ T-cell percentages were not altered in tumor (figure 5C). However, further analysis showed that IACS-010759 decreased radiation-induced T_regs_ (p=0.0076, XRT alone compared with XRT combined with IACS-010759; figure 5D). IACS-010759 also increased Gzmb+ T cells compared with control (p=0.015), and the combination of XRT with IACS-010759 significantly increased Gzmb+ T cells relative to XRT alone (p<0.0001; figure 5E). Similar with PD-1-resistant model, data in sensitive model also indicated that IACS-010759 decreased radiation-induced T_regs_ (p=0.026, XRT alone compared with XRT combined with IACS-010759; online supplementary figure 6A). IACS-010759 also increased Gzmb+ T cells compared with control (p=0.031), and the combination of XRT with IACS-010759 significantly increased Gzmb+ T cells relative to XRT alone (p=0.04; online supplementary figure 6B).

Collectively, these findings suggested that although IACS-010759 does not harm CD45+, CD4+, and CD8+immune cells, it may not be potent enough on its own to promote an abscopal response with XRT. This led us to evaluate whether adding IACS-010759 to XRT and PD-1 checkpoint inhibition would augment the abscopal effect.

### IACS-010759 increases the abscopal responses induced by XRT and PD-1 inhibition

**Figure 6:**
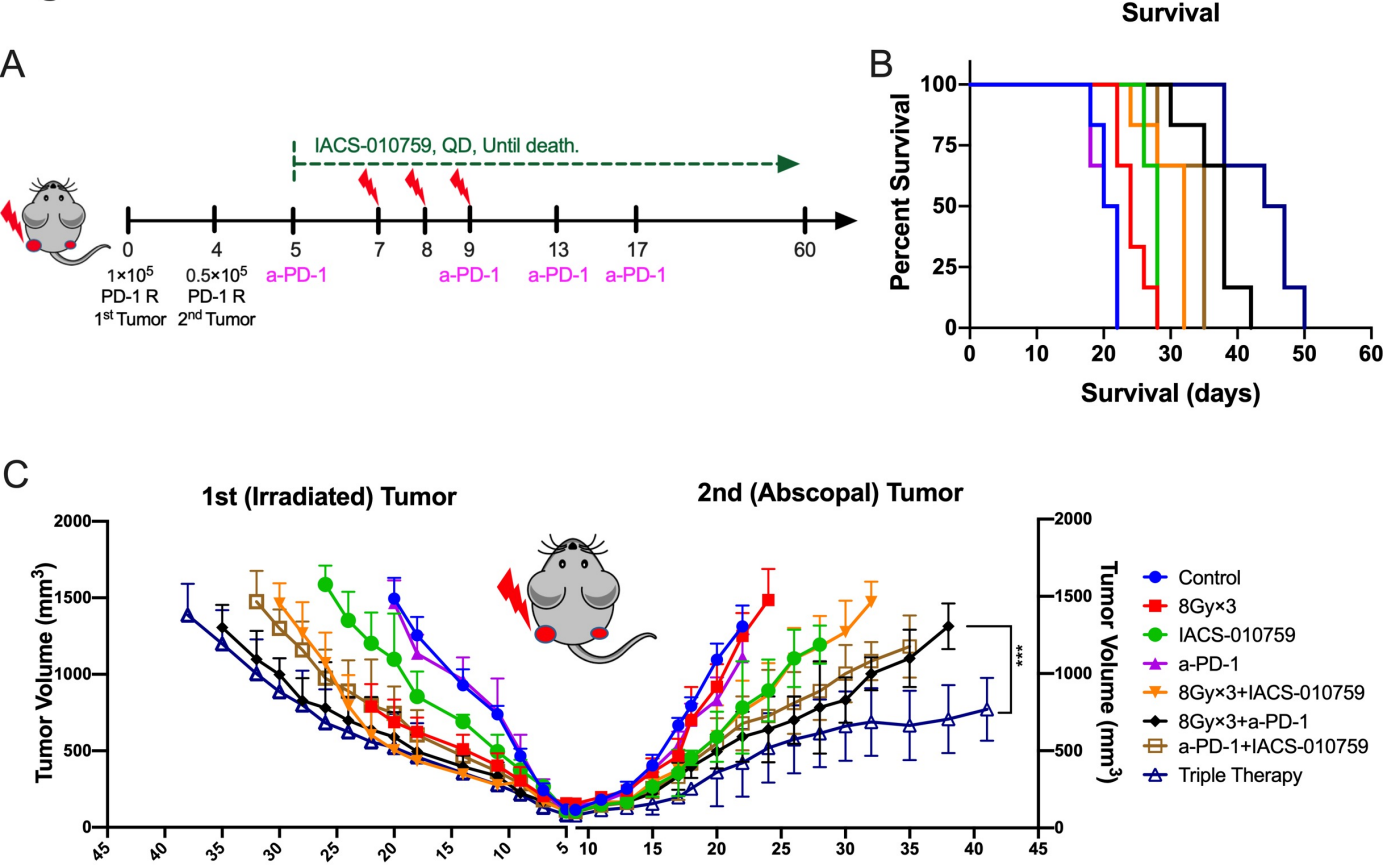
IACS-010759 induces stronger abscopal responses and prolongs survival when added to radiotherapy (XRT) plus anti-PD-1 therapy. (A) Schematic diagram for the two-tumor model: 1×10^5^ PD-1-resistant 344-SQ cells were injected into the left leg to establish the primary (irradiated) tumor on day 0 and 0.2×10^5^ cells were injected into the right leg to establish the abscopal (unirradiated) tumor (n=6 mice per group). Radiation was administrated on days 7, 8, and 9; anti-PD-1 was intraperitoneally injected on days 5, 9, 13, and 17; IACS-010759 was given orally from day 5 until death. (B) IACS-010759 plus anti-PD-1 plus XRT significantly prolonged survival compared with XRT plus anti-PD-1 (log-rank test, p=0.001). (C) Adding IACS-010759 to XRT plus anti-PD-1 boosted abscopal response (two-way ANOVA, p=0.0001). Data are presented as mean with standard deviation (*p<0.05, **p<0.01, **p<0.01, ****p<0.0001). ANOVA, analysis of variance.

To explore the abscopal effect, the PD-1-resistant two-tumor model in vivo was established as shown in figure 6A. Adding IACS-010759 significantly prolonged survival compared with XRT (8 Gy×3) plus anti-PD-1 without IACS-010759 (p=0.001; figure 6B). Furthermore, IACS-010759 combined with XRT seemed to overcome PD-1 resistance and activate antitumor immunity, significantly enhancing abscopal responses compared with XRT plus anti-PD-1 (p=0.0001; figure 6C).

## Discussion

The current study demonstrated that our PD-1-resistant 344-SQ model was characterized by increased OXPHOS at baseline and after XRT. We further found that IACS-010759, a novel OXPHOS inhibitor, might be preferentially beneficial to PD-1-resistant NSCLC, especially in combination with XRT. Moreover, the combination of XRT, anti-PD-1, and OXPHOS inhibition represented a promising approach to promote abscopal responses in PD-1-resistant disease.

In the current study, we found that the PD-1-resistant model was characterized by a more aerobic phenotype than the PD-1-sensitive model. This finding suggests that differential oncocellular metabolism may be associated with distinct immune microenvironments and differential response to immunotherapy. This notion is supported by recent results of Najjar *et al*, who demonstrated that OXPHOS may be a barrier to response to anti-PD-1 therapy in metastatic melanoma.^16^ The degree of mitochondrial OXPHOS likely varies across patient populations, even among those with similar histologic characteristics. The principle for this hypothesis is similar to that underlying the general lack of effectiveness of immunotherapy for driver mutation-positive NSCLC, 16 whereas inhibition of mitochondrial OXPHOS can increase TKI sensitivity, indicating that NSCLC with driver mutations may have distinct metabolic profiles that may associate with immunotherapy resistance.^17 18^

Many reviews indicated that XRT could increase the treatment efficacy of immunotherapy by reprogramming tumor microenvironment.^19 20^ Our previous study showed that XRT could induce type I IFN production, upregulated MHC-I expression, and restored response to anti-PD-1 in our anti-PD-1-resistant model.^9^ Although XRT can overcome PD-1 resistance in NSCLC,^21–24^ XRT seems to be a double-edged sword to the immune system. Despite releasing tumor-associated antigens and chemokines, recruiting activated cytotoxic lymphocytes, and inducing an abscopal antitumor response, XRT also increases T_regs_, myeloid-derived suppressor cells, and M2 tumor-associated macrophages.^21^ Moreover, we also found that XRT is associated with decreased glycolysis and increased OXPHOS in the PD-1-resistant model, which is known to increase hypoxia, a well-recognized correlate with radiation resistance.^25 26^ Actually, another study also proved the similar phenotype, which demonstrated that XRT could switch glycolysis to mitochondrial OXPHOS: XRT induces the localization of mTOR from cytosol to mitochondrial surface where it inhibits the activity of Hexokinase II. The mitochondrial OXPHOS increased mTOR and decreased glycolysis. At the same time, the mTOR-mediated HK II inhibition will decrease mitochondrial suppression, enhancing mitochondrial OXPHOS.^27–29^ Despite these negative effects of XRT, we found that IACS-010759 (in addition to inhibiting OXPHOS) can decrease radiation-induced T_regs_ and increase Gzmb+CD8+ T cells. This indicates that IACS-010759 can both address radiation-induced immunosuppression and also further boost activation of antitumor immunity.

The abscopal effect is uncommon, and a major focus of ongoing research is to explore strategies to make this event a more common phenomenon. Previous data have shown that combining high-dose XRT with anti-PD-1 therapy could overcome anti-PD-1 resistance and induce this effect.^8^ Because XRT is also associated with a change in metabolism that could contribute to further resistance, we hypothesized that combined treatment with XRT and IACS-010759 could produce abscopal responses to inhibit the growth of oligometastases. Although this did not occur for some of our dosing schemes (12 Gy×3 and 8 Gy×3) when combined with IACS-010759 alone, the reason was not related to CD45+, CD4+, and CD8+ immune cell destruction, which found to be the most important factors to induce abscopal response. We thus posit that although IACS-010759 has positive effects in activating antitumor immunity, it may simply not be efficacious enough to induce abscopal responses by itself. As a result, we then surmised that triple therapy with IACS-010759, XRT, and anti-PD-1 could help boost antitumor immunity, which our data then supported.

**Figure 7:**
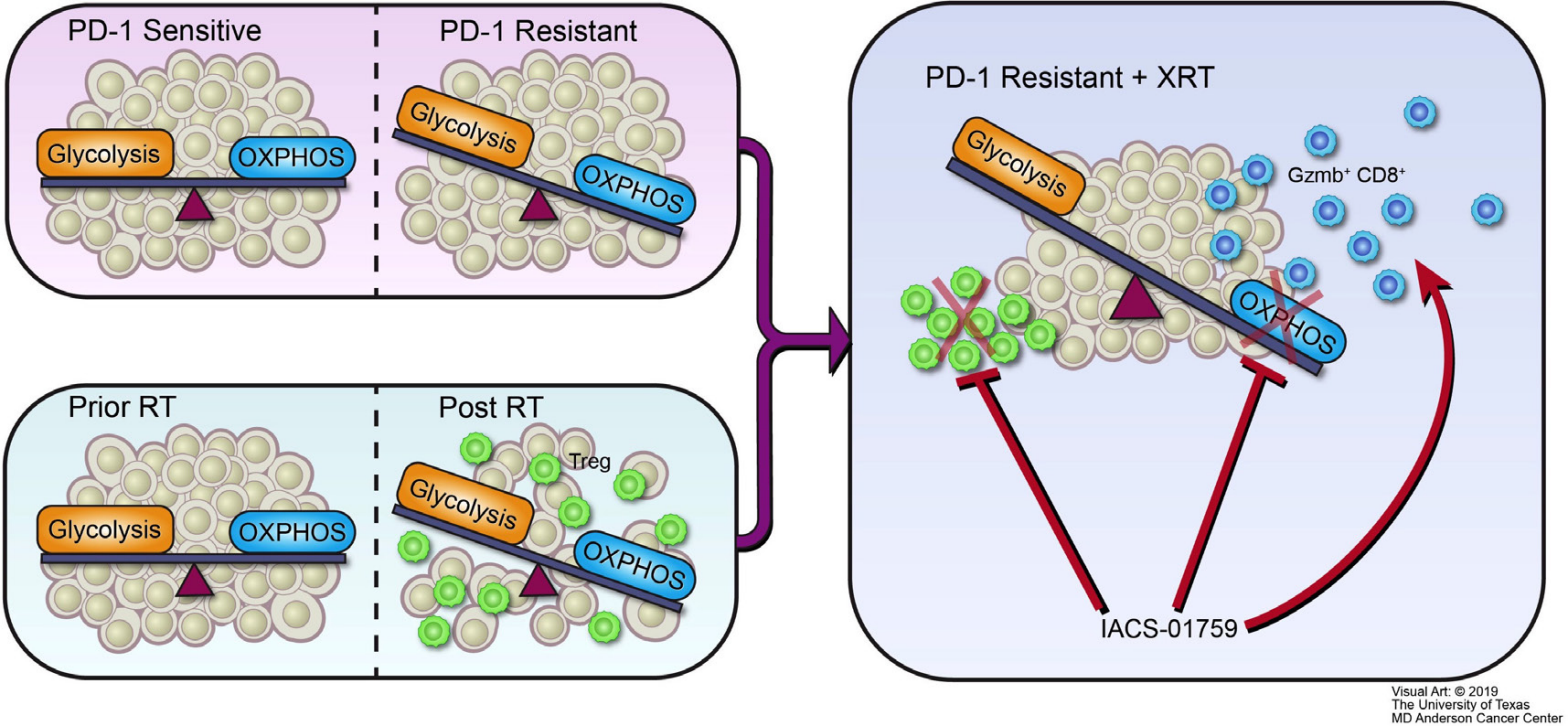
Schematic illustration. Oxidative phosphorylation (OXPHOS) inhibition ameliorates radiation-induced immune suppression and synergistically works with radiotherapy (XRT) to activate antitumor immunity. (A) PD-1-resistant model, which relies more on mitochondrial OXPHOS and is characterized by a more aerobic phenotype than the PD-1-sensitive model. (B) Radiation induces a switch to OXPHOS and increases immunosuppressive regulatory T cells (T_regs_). (C) Although radiation makes PD-1-resistant tumors induce more T_regs_ and OXPHOS than in PD-1-sensitive tumors, the mitochondrial OXPHOS inhibitor IACS-010759 can counteract this negative effect. First, IACS-010759 blocks OXPHOS, alleviates hypoxia, overcomes radiation resistance, and inhibits tumor growth; second, IACS-010759 inhibits radiation-induced T_regs_; third, IACS-010759 increases Granzyme B+ CD8+ T cells to boost antitumor immunity.

In conclusion, we propose the following structure to summarize the major outcomes of this study (figure 7). Our PD-1-resistant model utilized OXPHOS to a significantly greater extent than the PD-1-sensitive model and XRT increased OXPHOS in vitro and in vivo. The OXPHOS inhibitor IACS-010759 may be preferentially beneficial to PD-1-resistant NSCLC, especially in combination with XRT, and can alleviate radiation-induced immunosuppression and increase antitumor immunity. Moreover, the combination of XRT, anti-PD-1, and OXPHOS inhibition represents a promising approach to promote abscopal responses in PD-1-resistant NSCLC.
