# Supplementary material for: Combination treatment with radiotherapy and a novel oxidative phosphorylation inhibitor overcomes PD-1 resistance and enhances antitumor immunity
Source: J Immunother Cancer. 2020 Jun 24;8(1):e000289. doi: 10.1136/jitc-2019-000289 (PMC7319777; doi:10.1136/jitc-2019-000289)
Supplement: Supplementary data [file jitc-2019-000289supp001.pdf]

Supplemental Figure 1

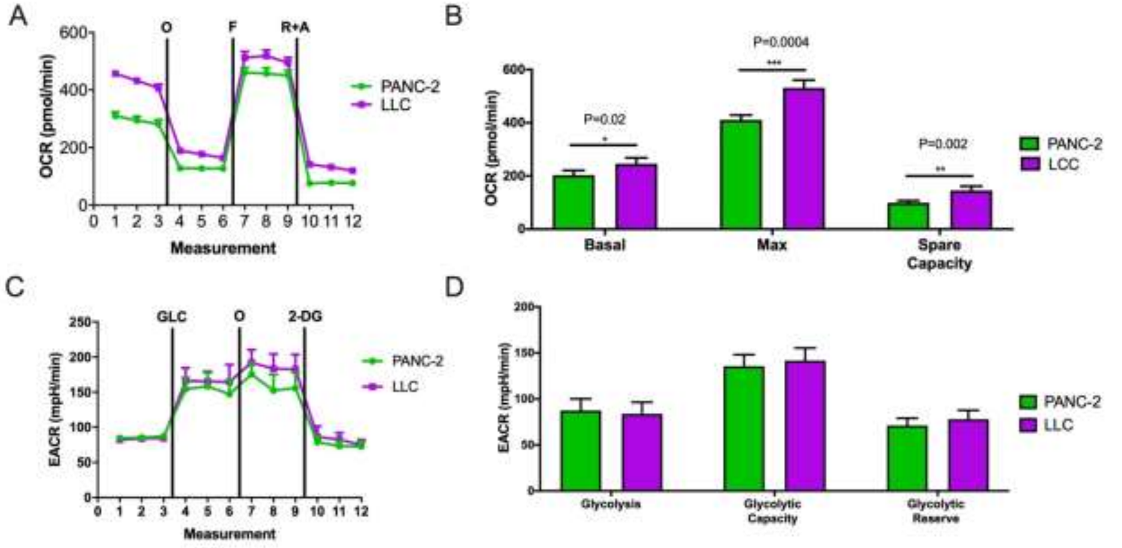

Supplemental Figure 1. LLC cell line (PD-1 resistant) relies more on OXPHOS comparing to PANC-2 cell line (PD-1 sensitive). (**A**)The Seahorse Mito Stress Test was performed to acquire baseline, oligomycin-inhibited, FCCP-activated, and rotenone/antimycin-inhibited OCR values. (**B**)Basal respiration (P = 0.02), maximal respiration (P =0.0004), and spare respiratory capacity (P = 0.002) were significantly higher in the LLC cell line (PD-1–resistant) than in the PANC-2 cell line (PD-1–sensitive). (**C**) The Seahorse Glycolysis Stress Test captured baseline, glucose-stimulated, oligomycin-activated, and 2-deoxyglucose-inhibited ECAR values in these two models. (**D**) Glycolysis and glycolytic capacity did not differ between LLC and PANC-2 cell lines in glycolysis, glycolytic capacity and glycolytic reserve (P > 0.05, respectively).

Supplemental Figure 2

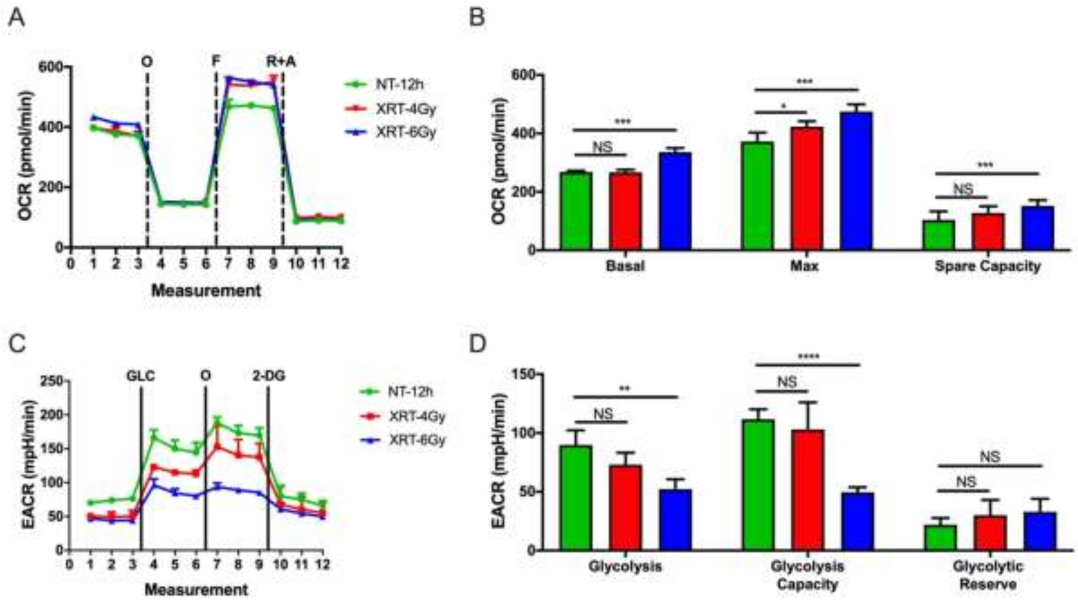

Supplemental Figure 2. Radiotherapy (4Gy and 6Gy) increases oxidative phosphorylation (OXPHOS) and decreases glycolysis 12 hours after XRT in the PD-1–resistant model. (A) Seahorse Mito Stress Test analysis showing oligomycin-inhibited (O), FCCP-activated (F), and rotenone/antimycin-inhibited (R+A) oxygen consumption rate (OCR) in the PD-1–resistant cell line 12 hours after XRT. **(B)** XRT significantly increased basal respiration (P = 0.0005 in the 6 Gy group), maximal respiration (t-test, P = 0.011 in the 4 Gy group; P < 0.0001 in the 6 Gy group), and spare respiratory capacity (t-test, P = 0.019 in the 6 Gy group) compared with control (n = 4 wells/group). **(C)** Seahorse Glycolysis Stress Test analysis showing glucose-stimulated (GLC), oligomycin-activated (O), and 2-deoxyglucose-inhibited (2-DG) extracellular acidification rate (ECAR) in the PD-1–resistant cell line 12 hours after XRT. **(D)** XRT decreased glycolysis (t-test, P = 0.0003 in the 6 Gy group) and glycolytic capacity (t-test, P < 0.0001 in the 6 Gy group) compared with control (n = 4 wells/group).

Supplemental Figure 3

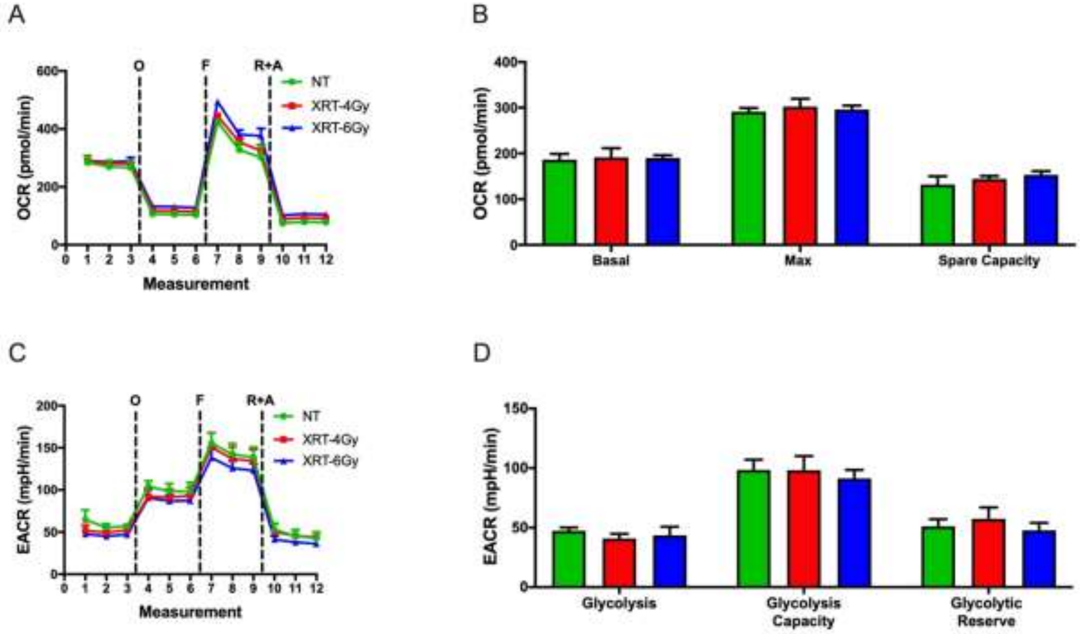

Supplemental Figure 3. Seahorse analysis to test mitochondrial and glycolysis stress after 12 hour XRT with 4Gy or 6Gy in the PD-1–sensitive 344-SQ model. In contrast to the PD-1 resistant model, no significant differences in OXPHOS **(A)** or glycolysis **(B)** were observed with irradiation.

Supplemental Figure 4

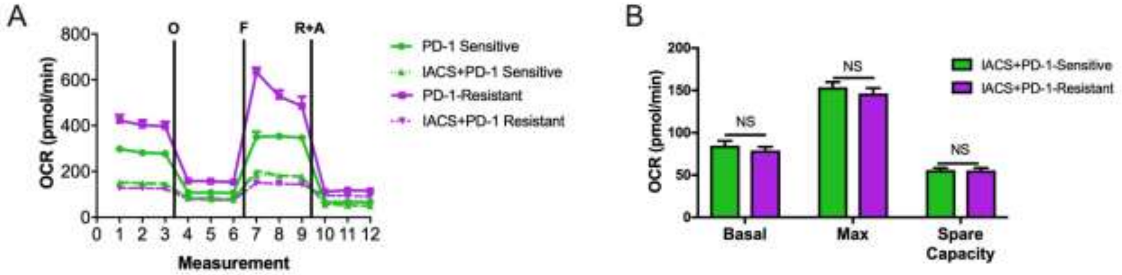

Supplemental Figure 4 IACS-010759 inhibited OXPHOS activity effectively in PD-1 sensitive and resistant model. (**A**) Bioenergetics stress tests of PD-1 sensitive and PD-1 resistant cell lines following Vehicle or IACS-010759 treatment (50nM) for 12h in vitro. Oligomycin- inhibited ("O"), FCCP-activated ("F") and Antimycin/Rotenone inhibited ("A&R") OCR levels are indicated. (**B**) After IACS-010759 treatment 12h, PD-1 sensitive and resistant cell lines showed similar basal respiration ($P = 0.74$), maximal respiration ($P = 0.62$), and spare respiratory capacity ($P = 0.9$).

Supplemental Figure 5

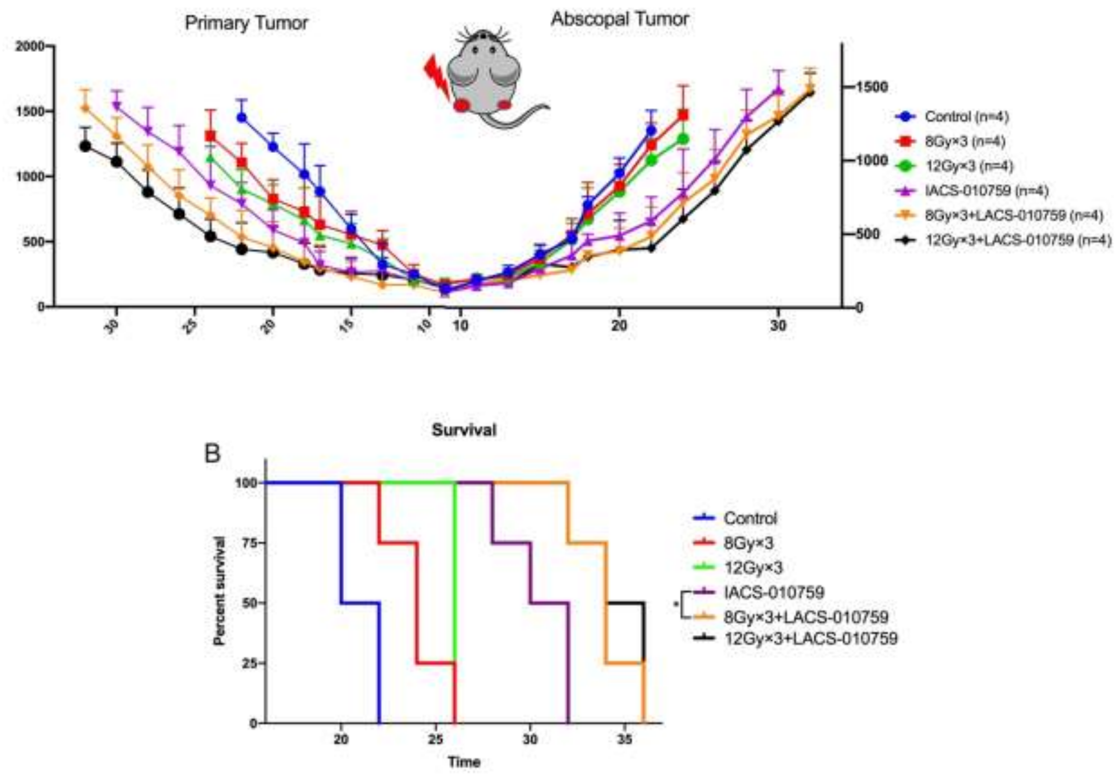

Supplemental Figure 5. Radiotherapy (XRT) combined with IACS-010759 did not induce abscopal responses but significantly prolonged mouse survival. (A) Neither 8 Gy × 3 nor 12 Gy × 3 XRT combined with IACS-010759 induced an abscopal response (two-way ANOVA). **(B)** The combination of XRT with IACS-010759 significantly increased mouse survival compared with IACS-010759 alone (log-rank test, P = 0.028 for 8 Gy × 3 and P = 0.014 for 12 Gy × 3).

Supplemental Figure 6

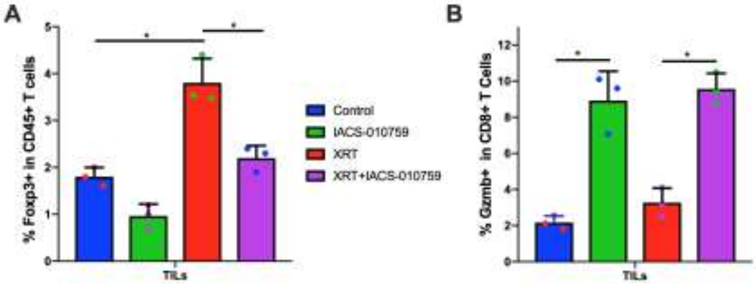

Supplemental Figure. 6 IACS-010759 decreased Tregs and increased Gzmb^{+} CD8^{+} T cells. (A) Similar with PD-1 resistant model, data in sensitive model also indicated that IACS-010759 decreased radiation-induced Tregs (P = 0.026, XRT alone compared with XRT combined with IACS-010759). (B) IACS-010759 also increased Gzmb+ T cells compared with control (P = 0.031), and the combination of XRT with IACS-010759 significantly increased Gzmb+ T cells relative to XRT alone.
